# Supraclavicular lymphadenopathy: an unexpected consequence of breast implant rupture

**DOI:** 10.1093/jscr/rjaf308

**Published:** 2025-05-13

**Authors:** Rasheed Elayyan, Rebaz Abdullah, Tani Fasih

**Affiliations:** University of East Anglia, Norwich Research Park, Norwich, Norfolk, NR4 7TJ, United Kingdom; Gateshead Health NHS Foundation Trust, Queen Elizabeth Ave, Gateshead NE9 6SX, United Kingdom; Gateshead Health NHS Foundation Trust, Queen Elizabeth Ave, Gateshead NE9 6SX, United Kingdom

**Keywords:** breast implants, supraclavicular lymphadenopathy, cervical lymphadenopathy, breast implant rupture

## Abstract

Supraclavicular lymphadenopathy is a rare manifestation of silicone lymphadenopathy, a complication arising from breast implant rupture. We present a case of a 39-year-old woman with a history of bilateral breast augmentation using Poly Implant Prothèse implants who developed left breast swelling and progressive supraclavicular lymphadenopathy. Imaging revealed rupture of the left implant with silicone migration to supraclavicular lymph nodes, confirmed by the characteristic “snowstorm” sign on ultrasound. Surgical intervention involved bilateral implant removal and partial capsulectomy, with histopathological evaluation showing benign findings. Silicone lymphadenopathy, though typically associated with axillary nodes, can extend to cervical region posing diagnostic challenges. This case highlights the importance of considering silicone lymphadenopathy in patients with unexplained cervical lymphadenopathy and a history of breast implants. Diagnostic imaging and patient-centered management strategies remain crucial in addressing this rare but significant condition.

## Introduction

Cervical lymphadenopathy is mostly attributed to local infectious or malignant process within its field of drainage. Cervical lymph nodes, including the supraclavicular nodes, drain regions such as the head, neck, breast, and upper abdomen, making them potential sites for malignancies originating from any of these areas [1]. For example more than a third (37.5%) of chest pathologies have the potential to metastasize to the cervical lymph nodes [2].

Breast augmentation is the most common cosmetic surgical procedure in the United Kingdom, with around 7000 cases recorded in 2022 [3]. The history of breast implants for augmentation began with the first silicone gel breast implant by Cronin and Gerow in 1964, marking the modern era [4]. This early implant was a thin-shelled device filled with low-viscosity silicone. Over the years, silicone implants evolved, with various brands and designs entering the market. However, Poly Implant Prothèse (PIP) implants were withdrawn in 2010 due to the use of industrial-grade silicone, which had a rupture rate up to 30 times higher than other implants [5].

Silicone lymphadenopathy, which occurs as a foreign body reaction to silicone, is a well-known complication of breast augmentation with silicone-based materials. It arises when silicone particles migrate to the regional lymph nodes. This can happen due to implant rupture or the leakage of tiny amounts of silicone gel through the outer shell, a process called “gel bleed.” Most cases involve lymphadenopathy in the axillary or mediastinal regions, while reports of silicone-associated lymphadenopathy in the cervical region are very rare [6, 7]. The incidence of silicone lymphadenopathy is more common in women with (PIP) implants due to the known increased rates of rupture [8].

To our knowledge, there are only a limited number of reported cases of silicone cervical lymphadenopathy. We are reporting a case of supraclavicular silicone lymphadenopathy attributed to a breast PIP implant rupture. This case contributes to the limited evidence of cervical silicone lymphadenopathy and highlights the importance of considering it in the deferential diagnosis when evaluating neck lumps in patients with a history of silicone breast implants.

## Case presentation

### Patient presentation

A 39-year-old woman with a history of bilateral breast augmentation 16 years prior presented with progressive left breast swelling over 1 week. Examination revealed left breast asymmetry, with marked enlargement and firmness compared to the right. Palpable left axillary lymph nodes were noted. The patient denied fever, pain, systemic symptoms, or prior breast issues and reported no significant comorbidities.

### Initial investigations

**Figure 1:**
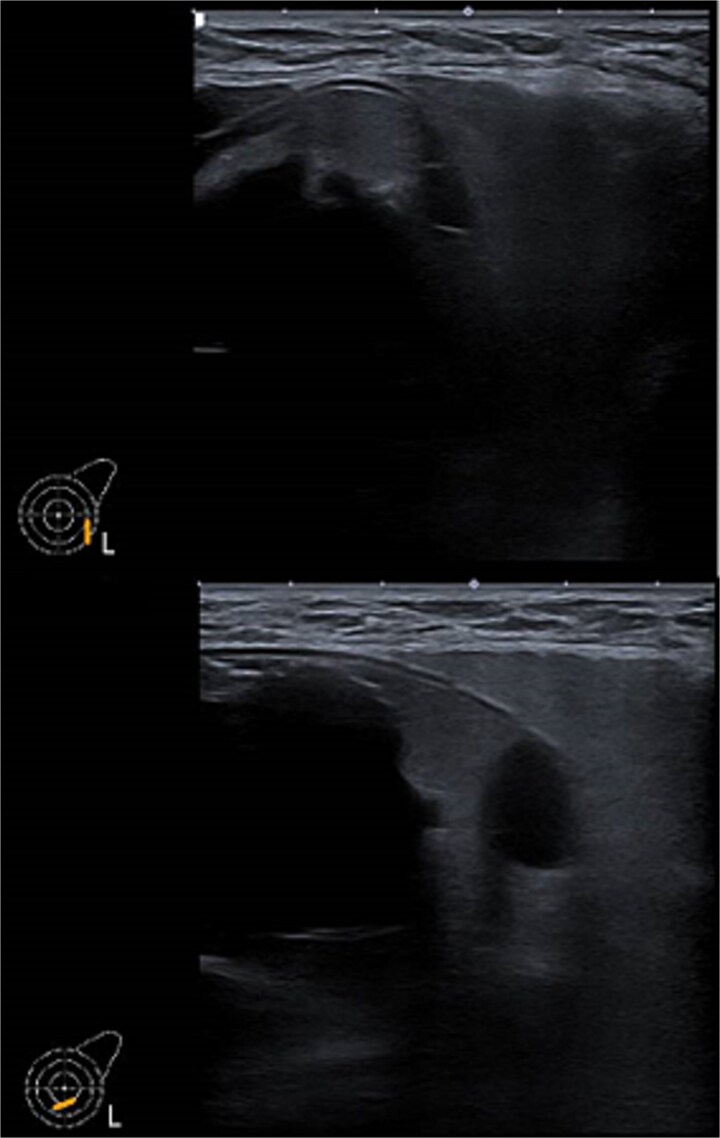
Ultrasound of the left breast showed rupture of the left breast implant with intracapsular and extracapsular leakage, surrounded by echogenic peri-implant fluid.

**Figure 2:**
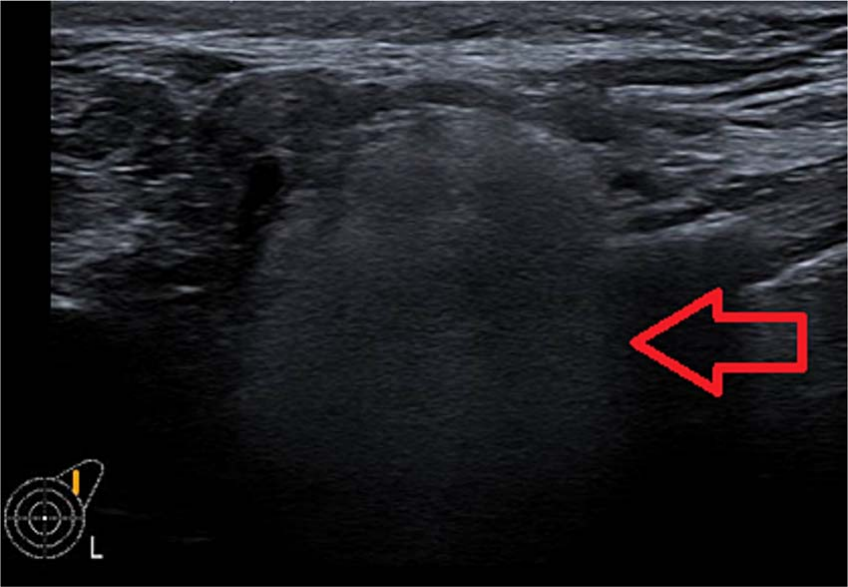
Ultrasound of the left axilla showed the typical appearance of the lymph node siliconoma.

- *Ultrasound of left breast and axilla*: Revealed rupture of the left implant with intracapsular and extracapsular leakage, along with echogenic peri-implant fluid. Enlarged axillary lymph nodes were visualized (Figs 1 and 2).
- *Fluid aspiration*: Approximately 180 mL of serous-like fluid was aspirated. Cytology showed bland adipocytes, lymphoid cells, and debris without malignancy.

The patient was scheduled for bilateral implant removal with or without capsulectomies.

### Subsequent course

**Figure 3:**
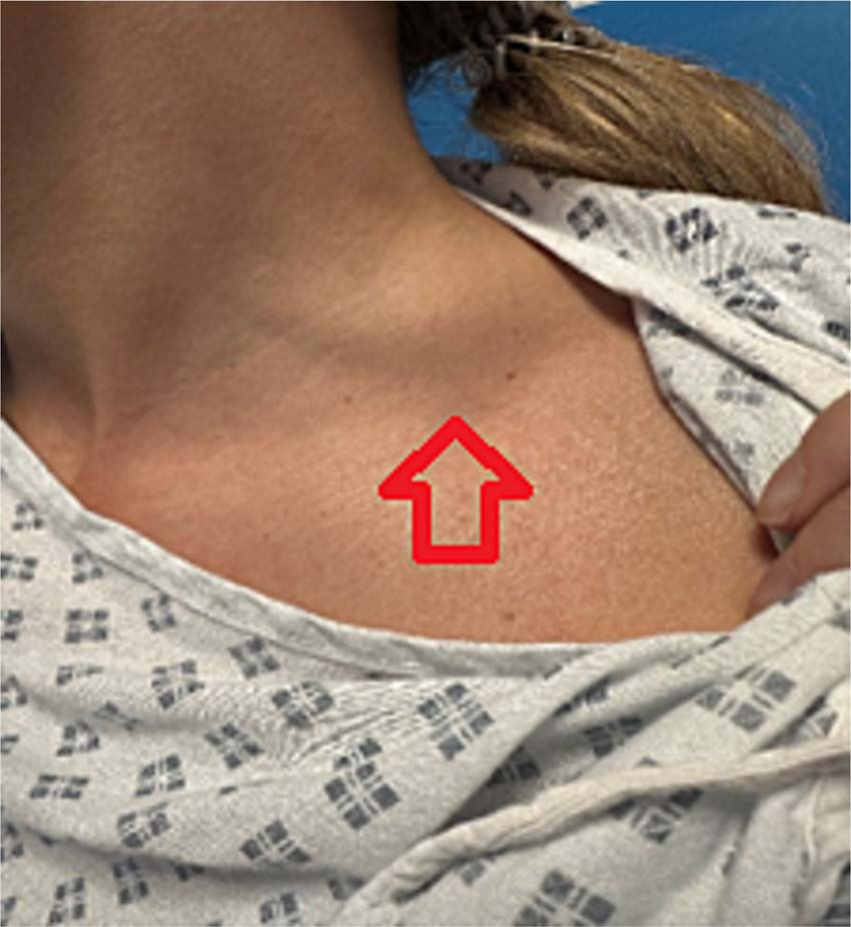
Newly developed supraclavicular lump.

**Figure 4:**
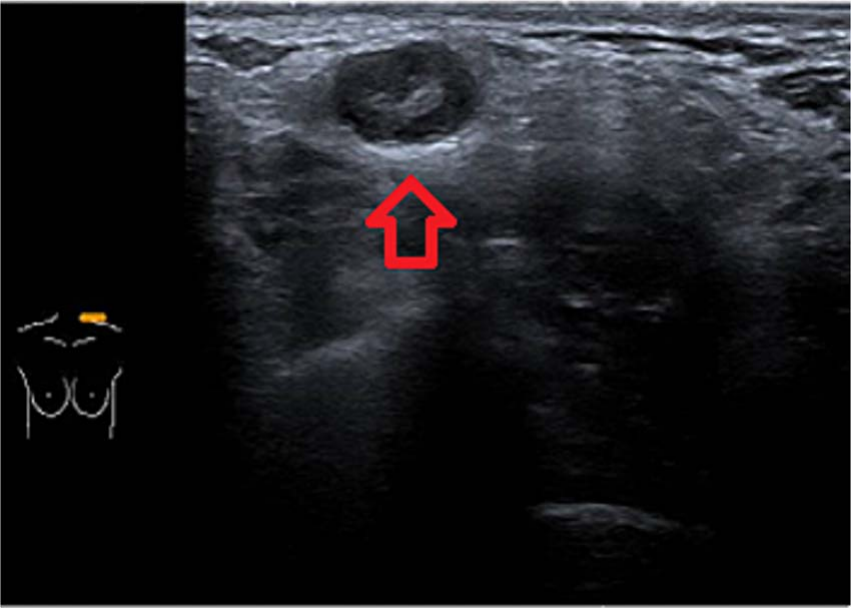
Typical snowstorm appearance of supraclavicular silicone lymphadenopathy.

One week later, the patient reported a new lump in the left supraclavicular area. Examination revealed a 2 × 3 cm, nontender, mobile mass, confirmed by ultrasound to be silicone deposition (Figs 3 and 4). Increased breast swelling and lymphadenopathy were attributed to implant rupture and silicone migration.

### Emergency presentation

**Figure 5:**
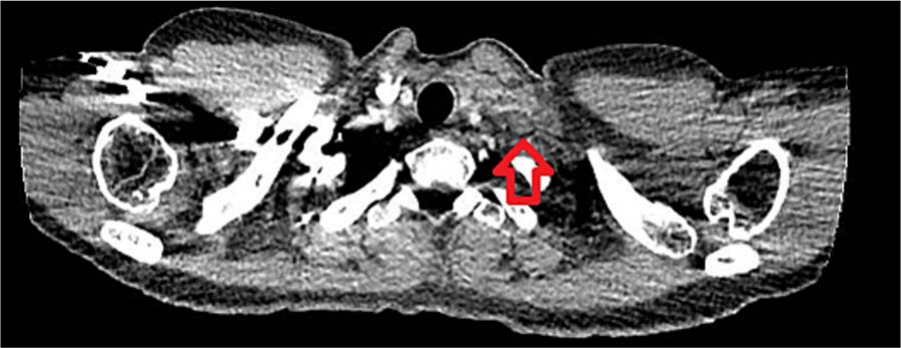
CT thorax showed a 25 × 9 mm supraclavicular mass.

The patient presented to the emergency department the same day with shortness of breath. Investigations were normal except for a mildly elevated D-dimer. A CT pulmonary angiogram excluded pulmonary embolism but confirmed the supraclavicular mass (Fig. 5). The patient was reassured and referred back to the breast clinic.

### Surgical management

The patient underwent bilateral implant removal 2 weeks later.

**Figure 6:**
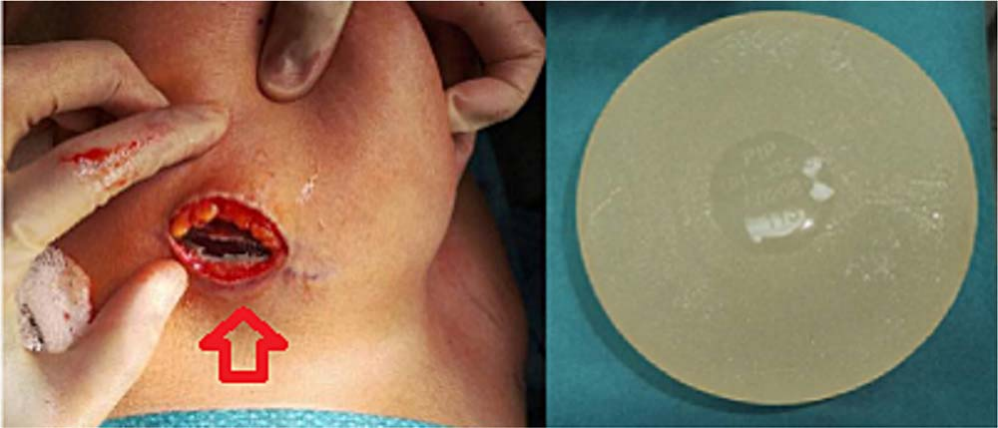
Intact right breast PIP implant.

**Figure 7:**
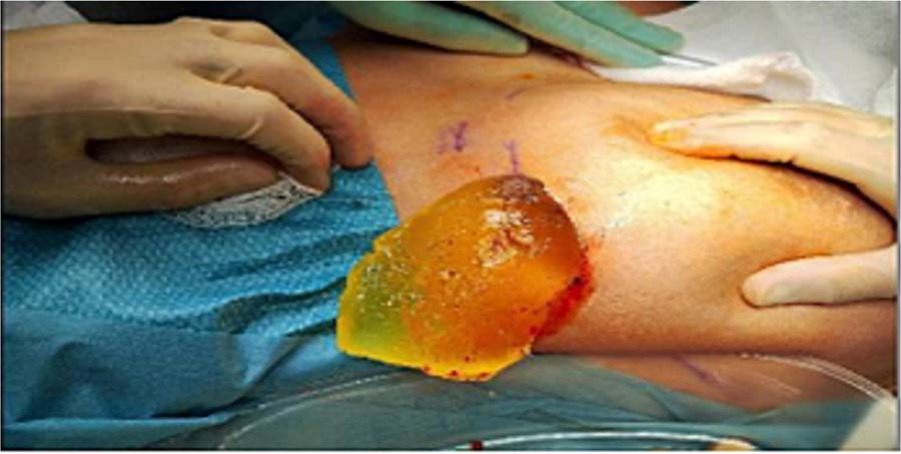
Ruptured left breast PIP implant.

- Intraoperative findings:
  - Right implant: Intact, without capsular contracture or nodularity (Fig. 6).
  - Left implant: Ruptured, with a thin, smooth capsule (Fig. 7).

A partial capsulectomy was performed on the left side due to poor dissection planes. Histopathology revealed benign features.

## Discussion

Breast implants are regarded as a safe surgical option with minimal associated complications. Recent innovations in their design and new technologies have improved their safety, reliability, and acceptance [9]. Silicone lymphadenopathy is a breast implant complication characterized by the migration of silicone to surrounding soft tissues or lymph nodes. Silicone is transported to nearby lymph nodes by macrophages, where it initiates a foreign body reaction. This reaction causes chronic inflammation, eventually leading to lymphadenopathy [6].

A recent systematic review of 190 reported silicone lymphadenopathy areas showed that axillary lymphadenopathy was the most common, with 136 cases representing 72% of the cases, internal mammary lymphadenopathy in 40 case representing 21% of the cases, cervical/supraclavicular lymphadenopathy in 36 cases representing 19% of the cases, mediastinal in 24 cases representing 13% of the cases, and in 43 cases multiple nodal regions were involved representing 23% of the cases [10].

Imaging is a vital first step in the diagnostic evaluation of patients with lymphadenopathy. Ultrasound is the primary tool for diagnosing silicone lymphadenopathy, while additional imaging techniques, including MRI, CT, and mammography, can offer further insights for confirmation and detailed assessment of lymphadenopathy [11]. The sensitivity and specificity of using ultrasound to diagnose silicone lymphadenopathy by identifying the snowstorm sign have been reported as 87.5% and 100%, respectively [11]. In our case, the ultrasound typical snowstorm appearance was enough to make the diagnosis. Even though if any clinical concern about malignant or infectious process, cytological or histological evaluation is mandatory to confirm the diagnosis. Also in patients with previously treated breast cancer and having the implant as reconstructive measure; concerns about disease recurrence should be carefully considered.

The implant’s integrity should always be assessed, considering surgical intervention if rupture is identified. For enlarged lymph nodes, the key decision is excision or monitoring, made collaboratively with the patient. In our case, as the lymphadenopathy was asymptomatic with no compressive symptoms or recurrent inflammatory flare-ups, monitoring was chosen.

## Conclusion

This case highlights the importance of considering silicone lymphadenopathy as a possible differential diagnosis in patients with supraclavicular adenopathy and a history of breast implants, particularly following implant rupture and when other causes are not identified. Otolaryngologists should take this pathology into account when there is diagnostic uncertainty in cases of isolated lymphadenopathy in the neck.

## References

[1] Nikolaev NN, Yankov YG. Lymph nodes of the head and neck: in normal and pathological conditions. Varna Med Forum 2023;12:69–74. 10.14748/vmf.v12i1.9034

[2] Van Overhagen H, Brakel K, Heijenbrok MW, et al. Metastases in supraclavicular lymph nodes in lung cancer: assessment with palpation, US, and CT. Radiology 2004;232:75–80. 10.1148/radiol.2321030663 15166326

[3] British Association of Aesthetic Plastic Surgeons (BAAPS) . BAAPS National Audit 2022. BAAPS, 2023. https://baaps.org.uk/media/press_releases/1872/cosmetic_surgery_boom (1 May 2023, date last accessed)).

[4] Cronin TD, Gerow FJ. Augmentation mammaplasty: a new ‘natural feel’ prosthesis. In: Grabb WC, Smith JW (eds), Transactions of the Third International Congress of Plastic Surgery. Amsterdam: Excerpta Medica, 1964.

[5] Department of Health . Poly Implant Prothése (PIP) Breast Implants: Final Report of the Expert Group. Leeds: DH, 2012.

[6] Borghol K, Gallagher G, Skelly BL. Silicone granuloma from ruptured breast implants as a cause of cervical lymphadenopathy. Ann R Coll Surg Engl 2016;98:e118–20. 10.1308/rcsann.2016.0166 27167311 PMC5209993

[7] Omakobia E, Porter G, Armstrong S, et al. Silicone lymphadenopathy: an unexpected cause of neck lumps. J Laryngol Otol 2012;126:970–3. 10.1017/S0022215112001089 22672792

[8] Zambacos GJ, Molnar C, Mandrekas AD. Silicone lymphadenopathy after breast augmentation: case reports, review of the literature, and current thoughts. Aesthetic Plast Surg 2013;37:278–89. 10.1007/s00266-012-0025-9 23354761

[9] Accurso A, Rocco N, Feleppa C, et al. Spread of silicone to axillary lymph nodes after high cohesive gel silicone implant rupture. Plast Reconstr Surg 2008;122:221e–2e. 10.1097/PRS.0b013e31818d221f 19050500

[10] Pelegrina Perez TC, Desai A, Tadisina KK, et al. Prevalence, clinical characteristics, and management of silicone lymphadenopathy: a systematic review of the literature. J Plast Reconstr Aesthet Surg 2024;90:76–87. 10.1016/j.bjps.2024.01.011 38364672

[11] Manzil FFP, Bhambhvani PG. 18F-FDG PET/CT unveiling of implant rupture and clinically unsuspected silicone granuloma in treated breast cancer. J Nucl Med Technol 2018;46:394–5. 10.2967/jnmt.118.211979 30076249

